# Supplementary material for: Towards unified AI-driven fracture mechanics: the extended deep energy method (XDEM)
Source: Nat Commun. 2026 Aug 15;17:8492. doi: 10.1038/s41467-026-76748-1 (PMC13484471; doi:10.1038/s41467-026-76748-1)
Supplement: Supplementary file 3 [file 41467_2026_76748_MOESM3_ESM.zip › pic/Results/Crack_propagation/Straight/l_0.015/Loss_convergence_2.pdf]

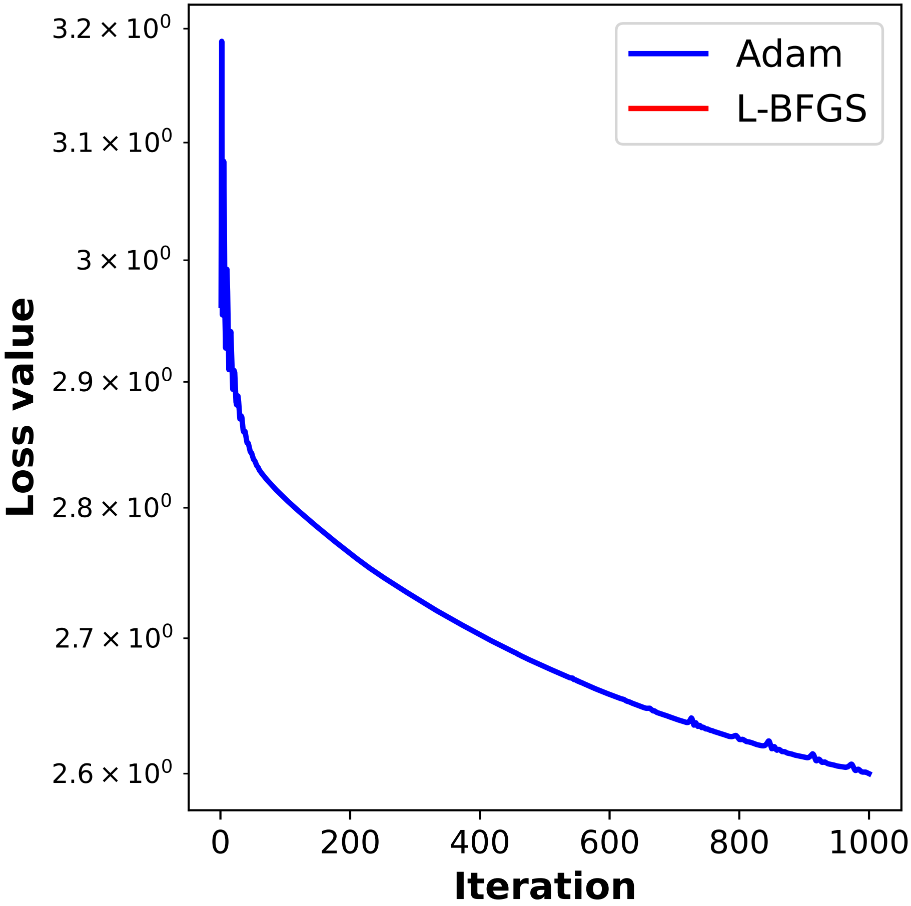
Adam
L-BFGS
Loss value
Iteration
3.2×10^0
3.1×10^0
3×10^0
2.9×10^0
2.8×10^0
2.7×10^0
2.6×10^0
0
200
400
600
800
1000
